# Towards an educational data literacy framework: enhancing the profiles of instructional designers and e-tutors of online and blended courses with new competences

**DOI:** 10.1186/s40561-021-00163-w

**Published:** 2021-09-17

**Authors:** Zacharoula Papamitsiou, Michail E. Filippakis, Marilena Poulou, Demetrios Sampson, Dirk Ifenthaler, Michail Giannakos

**Affiliations:** 1 Department of Computer Science, Norwegian University of Science and Technology, Sem Sælands vei 9, IT-Bygget, Gløshaugen, 7034 Trondheim, Norway; 2 University of Piraeus, Piraeus, Greece; 3 University of West Attica, Athens, Greece; 4 Curtin University, Curtin, Australia

**Keywords:** Educational data literacy, Competence profiles, Framework, Evaluation study

## Abstract

In the era of digitalization of learning and teaching processes, Educational Data Literacy (EDL) is highly valued and is becoming essential. EDL is conceptualized as the ability to collect, manage, analyse, comprehend, interpret, and act upon educational data in an ethical, meaningful, and critical manner. The professionals in the field of digitally supported education, i.e., Instructional Designers (IDs) and e-Tutors (eTUTs) of online and blended courses, need to be ready to inform their decisions with educational data, and face the upcoming data-related challenges; they need to update and enhance their profiles with relevant competences. This paper proposes a framework for EDL competence profiles of IDs/eTUTs and evaluates the proposal with the participation of worldwide professionals (*N* = 210) with experience in digitally supported education. The evaluation aims at validating the proposal and assesses (a) the current EDL-readiness of IDs/eTUTs; and (b) the extent to which the framework captures and describes the essential EDL competences. The findings indicate that professionals are not EDL-competent yet, but the proposed dimensions and related competences are offering a solid approach to support EDL development.

## Introduction

The human ability to understand, learn from, and use data as part of everyday thinking and reasoning for solving real-world problems is synopsized under the term *Data Literacy* (DL) (Shields, 2005). As daily interactions with data become commonplace and individuals more frequently make judgments from data and decisions regarding the use of their own data (Mortier et al., 2014), DL is considered a life competence. *Competence* is defined as a set of skills, knowledge, and attitudes that are possessed or need to be acquired to perform an activity within a specific context, whereas performance may range from the basic level of proficiency to the highest levels of excellence (Sampson & Fytros, 2008). In the context of professional development, competence refers to one’s capacity to apply a set of related skills, knowledge, and attitudes for the successful performance of critical *job functions,* in the given profession. The set of competences needed for a particular profession are described in a *Competence Profile* (CP); a CP provides insight in the functioning of professionals within the specific job context and can be used as a starting point for the professional development within this context.

In professional online and blended learning and teaching contexts, CPs can be utilized for the design of professional development courses, as well as for constructing instruments for competence assessment or course accreditation (Zervas et al., 2014). In those contexts, two important job roles are recognized: (a) the Instructional Designer (ID), who is in charge of designing and developing (together with subject domain experts) the online courses supported by a particular Learning Management System (LMS); and (b) the e-Tutor (eTUT) who is involved in supporting the delivery of online courses through the LMS (Kang & Ritzhaupt, 2015). Recognizing the importance of those two job roles for designing and delivering effective online professional development programs, several competence frameworks have been proposed, validated, and adopted during the past decade (e.g., CEN, 2016; Mishra & Koehler, 2006; UNESCO, 2011; Wakefield et al., 2012).

However, those competence frameworks for both jοb roles face a common significant shortcoming: they primarily aim at upskilling professionals in terms of educational design concepts and processes, or generic ICT competences and digital literacy; they scarcely—if at all—accommodate emerging advancements in the field of digital learning related to the use of educational data analytics methods (Sergis & Sampson, 2017) and the need for *Educational Data Literacy* (EDL). In professional teaching and learning settings, EDL is conceptualized as one’s level of understanding of how to find, evaluate, and use data to inform teaching and learning (Hamilton et al., 2009; Mandinach et al., 2008). In line with this abstract yet generic description of EDL, a data literate education professional should possess the knowledge, skills, and competence to gather, analyse, and convey information and data in a way that informs and supports decision-making through all phases of the educational process. Henderson and Corry (2020) conducted the review of literature review on data literacy in K-12 teacher education, as it is reflected on 28 articles from 2010 to 2018, to gain a better understanding of the current state of data literacy research. The authors affirm that although the concept of data literacy has become more concrete, however there is still disagreement about the parameters of the construct. They prompt for additional targeted research to influence training practices for both teachers and educational leaders, to ensure the understanding of data collection and statistical techniques and how that data should be used to inform instruction. On a similar conclusion converged another recent review; Ndukwe and Daniel (2020) agree that teachers’ training on data literacy is sporadic and scarce and that it is essential to empower teachers with the necessary knowledge of analytics and data literacy. Doing so is expected to prevent the poor interpretation of analytics, which in turn could lead to ill-informed decisions that can significantly affect students. However, as shown in another review study, most approaches to educators’ data literacy tend to cover fragmented sets of abilities, mostly connected to management and address technical skills, with less emphasis on critical, ethical and personal approaches to datafication in education (Raffaghelli & Stewart, 2020). It has been argued that teachers are inundated with data, and their capacity to use data productively and responsibly is a salient but complex skillset (Kippers et al., 2018; Raffaghelli & Stewart, 2020). A recent study showed that an asynchronous online data literacy intervention for pre-service and in-service educators affected educators’ data-driven decision making self-efficacy and anxiety as well as the in-school implementation of data use practices (Reeves, & Chiang, 2019).

In the era of digitalization of education, and as the availability in educational data increases rapidly, we need to determine in detail what today’s education professionals, i.e., IDs and eTUTs need to be able to know and do (with respect to educational data), to design and implement professional development programs accordingly, and equip them with tools to use that data optimally and ethically. Capitalizing on the potential of educational data analytics to personalize the authentic learning experiences and provide in-depth evidence on learners’ performance and needs (Papamitsiou et al., 2020), it becomes apparent that the definition of *Educational Data Literacy Competence Profiles* (EDL CP) for the roles of IDs and e-TUTs of online and blended courses is an essential extension of existing competence profiles of these two roles in digital learning industry. With respect to the two key roles, and towards extending previous frameworks, (a) the notion of EDL needs to be explicitly defined; (b) its dimensions need to be specified; and (c) the associated competences should be clarified.

In addition to that, the EDL-readiness of IDs/eTUTs should be determined. ‘Readiness’ is a necessary condition for one to be able to do something well; in order to do something well, one must first be *ready to do it*. In other words, ‘readiness’ refers to the degree/level of IDs’/eTUTs’ competences to adopt data-driven aspects in educational decision making and signifies the momentum to invest on developing the relevant competences. This need is even more emphatic in times of educational disruption, such as the recent Covid-19 pandemic; the outbreak of Covid-19 led the governments worldwide to take drastic measures and impose restrictions that resulted in national schools and universities closure and switching to fully online education (Viner et al., 2020). The Covid-19 crisis catalysed the demand to support the mode of totally online education by making proper use of educational data and analytics and highlighted the urgency to pay attention to the development of education professionals’ EDL competences. Sánchez-Cruzado et al. (2021) acknowledge that it is “essential to focus on the specialized training of teachers, spotting their main weaknesses and looking into them, helping them to attain an adequate level of data literacy, in order to face the new educational paradigm successfully” (p. 26), i.e., the challenges posed by the pandemic.

Several frameworks have been proposed during the past decade for addressing issues related to data literacy competences—some of those frameworks originate from areas that are close to the educational domain (e.g., library studies), while others focus on data literacy for teaching (e.g., Mandinach & Gummer, 2016; Prado & Marzal, 2013; Ridsdale et al., 2015). Despite that these proposals aim to tackle the same/similar problem(s), the diversity in their approaches leads to heterogeneous competences descriptions that cannot be formally described and represented in a unified manner. Furthermore—and to the best of our knowledge—none of those frameworks focuses on the competence profiles of IDs/eTUTs of online and blended courses. This paper introduces a definition for EDL and proposes and validates a unified EDL CP framework for the two discrete job roles. The overarching research question is threefold:

***RQ: (a)*** *What are the dimensions and competence statements of a unified EDL CP framework for the roles of instructional designers and e-tutors of online and blended courses?* ***(b)*** *What is the current EDL readiness of instructional designers and e-tutors of online and blended courses?* ***(c)*** *Does the proposed framework represent all facets of EDL to describe the essential competences of instructional designers and e-tutors of online and blended courses?*

To answer to the research question, we reviewed selected articles and courses relevant to EDL concepts. The goal was to come up with a proposal for a framework that establishes the extension of existing frameworks with new competences for both job roles (i.e., ID/eTUT), and can be taken as a benchmark by any professional development initiative that exploits EDL competence-based online/blended courses. Next, we identified the EDL dimensions and/or statements across existing frameworks and we synthesized and classified the results from the environmental scan into key EDL dimensions and competence statements per dimension. We also critically reflected on the included educational data aspects based on our group’s background on data science. To validate the proposed EDL CP framework, a study was conducted with the participation of *N* = 210 professionals with experience in digitally supported education from higher education institutes and elearning industry enterprises. An online survey instrument was designed to measure participants’ perceptions regarding the current EDL readiness of IDs/eTUTs and the appropriateness of the proposed framework to map the profiles.

The next section reviews and synopsizes the related work on the existing frameworks and EDL initiatives, aiming at identifying EDL-relevant dimensions and core competence statements per dimension, and then outlines the proposed EDL CP framework. The third section presents the methodology employed in the empirical study conducted for the validation of the proposed framework, and the fourth section presents and elaborates on the research findings. Finally, the paper discusses the results and concludes with the implications and limitations of the research.

## Related work

We understand here as EDL Competence Framework (EDL CF) an instrument for the development or assessment of EDL competences according to a set of criteria. The instrument establishes descriptors of intertwined competences aimed at boosting the EDL of the specific target groups, i.e., ID/eTUT. In essence, the instrument will cover all dimensions of EDL and synthetically shape the competence profiles for IDs/eTUTs. Thus, we selected to review articles or reports which propose a systematisation or interpretation of EDL-related concepts, as well as online and blended (professional development and/or higher education) courses intended to address EDL, and to organize the findings in a structured model/framework. The bibliometric approach applied in this study, in order to develop the framework, was scoping review (Arksey & O'Malley, 2005), combining a literature review with environmental scan of related work. The purpose of a scoping review is to provide an overview of the available research evidence for answering broad questions—unlike systematic reviews that commonly select the information sources by requiring specific study types, imposing quality standards, and placing their emphasis on synthesizing data to address a specific research question. Scoping studies follow a five-stages methodology: (1) identify the research questions (here, the research question is stated in the Introduction section), (2) identify relevant studies, (3) select studies, (4) charting the data, and (5) collating, summarizing, and reporting the results.

The studies presented here have been identified and collected after an extensive and iterative search in international databases of authoritative academic resources and publishers. Scans of concepts related to EDL (i.e., *educational data literacy; educational data literacy framework; educational data literacy competences; data literacy; data literacy framework; educational data analytics use; use of educational data for teaching; use of educational data for instruction; use of educational data for instructional design; educational data-driven decision making; educational data-driven instructional design; educational data-driven courses; educational data usage for training*) were conducted in academic publication databases (i.e., Web of Science; ERIC; Scopus; Google scholar; Sciencedirect). In addition, simple Google search was performed for the identification of the professional development courses. As EDL is an emerging field, we also searched for grey literature and identified white papers. We noticed that courses and workshops, and associations and organizations reports were more recent and updated. The time frame of the search was bound from 2005 to 2018, when the emergence of data literacy has grown. We screened a total of 134 articles, reports, and courses, 38 of which met the criteria to be further reviewed, i.e., emphasis was given to works reporting on existing EDL or EDL-relevant competence profiles, especially the ones that provide detailed competence statements, as they are the core references to analyse. An important limitation concerned terminology issues, as the terms ‘competence’ and ‘literacy’ are used with different meaning in different contexts. After reviewing the selected literature, we identified five (5) major approaches for EDL competence frameworks (EDL-CFs) and twelve (12) professional development/higher education courses related to introducing or teaching EDL skills or fundamentals of learning analytics concepts and usage. The selection and inclusion of the EDL frameworks was based on explicit criteria: the framework should consist of discrete conceptual dimensions and cover the competences that correspond to each one of these dimensions. Optionally, yet in most cases, the research groups/authors proposed a set of tasks linked to these competences for elaborating on and reasoning the practical implications of the respective competences. Furthermore, the selection of the courses was based on the target audience with a focus on IDs/eTUTs. Non-statistical methods were used to analyse the collected resources, to conduct the charting of the data, to collate and summarize the results, to evaluate and interpret findings of the collected resources, and to build the EDL competence profiles.

### Environmental scan of existing EDL competence frameworks and courses

The 5 approaches for EDL-CFs and the twelve (12) professional development/higher education courses are briefly presented in this section.

More precisely, Ridsdale et al. (2015) proposed a framework for *Data Literacy Education*, along with best practices for teaching data literacy across disciplines. The framework consists of five dimensions: (1) conceptual framework, i.e., general knowledge and understanding about data and the uses and applications of data; (2) data collection, i.e., skills and knowledge related to data discovery and collection from multiple educational sources, ensuring quality of datasets; (3) data management, i.e., skills that relate to data organization, preservation, manipulation, curation and security; (4) data evaluation, i.e., skills related to data analysis, presentation, interpretation and to making instructional decisions from data; and (5) data application, i.e., knowledge and skills needed to share and cite data, to evaluate decisions based on data and to work with data in an ethical manner. The authors defined the core skills and competences that comprise data literacy, using a thematic analysis of the elements of data literacy described in peer-reviewed literature. The included terms are broadly defined and involve a variety of elements considered core to EDL. The competencies and their skills, knowledge, and expected tasks are organized under the top-level elements of the definition (data, collect, manage, evaluate, apply) and are categorized as conceptual, core, and advanced competencies.

With an emphasis on pertaining teachers’ data literacy skills, Mandinach and Gummer (2016) provided a broader definition of what they call *data literacy for teaching*: “the ability to transform information into actionable instructional knowledge and practices by collecting, analyzing, and interpreting all types of data (assessment, school climate, behavioral, snapshot, longitudinal, moment-to-moment, and so on) to help determine instructional steps. It combines an understanding of data with standards, disciplinary knowledge and practices, curricular knowledge, pedagogical content knowledge, and an understanding of how children learn” (p. 367). In line with this definition, their framework combines seven key knowledge areas and five data-use aspects. Particularly, the five data-use domains include: (1) identify problems and frame questions, i.e., identify the problem, topics, issues, or questions to be addressed; (2) use data, i.e., skills varying from identification of data sources to developing sound assessment design and implementation, and from understanding how to analyse data to articulating inferences and conclusions from the analysis; (3) transform data into information, i.e., moving data toward information and using the learning context to make meaning and inform decisions; (4) transform information into a decision, i.e., the instructional steps based on the inquiry cycle; and (5) evaluate outcomes, i.e., examine the impact of the decision making process.

Furthermore, Means et al. (2011) raised the issue that, in general and to that time, teacher training programs had not addressed data skills and data-informed decision-making processes. Working with external data system and measurement experts, the research group identified five dimensions of data literacy for teachers: (1) data location, i.e., finding relevant and available pieces of data in the data system; (2) data comprehension, i.e., understanding what the data signify; (3) data interpretation, i.e., figuring out what the data mean; (4) data use for instructional decision making, i.e., selecting an instructional approach to address the situation identified through the data; and (5) question posing, i.e., framing instructionally relevant questions that can be addressed by the data in the system. For evaluating these five dimensions, the research team collected data scenario responses (interviews) from individual teachers and small groups of school staff. The responses were transcribed and analyzed using a standard coding scheme, aiming at identifying strengths/weaknesses of each DL component.

In addition, Prado and Marzal (2013) were inspired by the general structure of information literacy standards and proposed a framework as reference for the inclusion of data literacy in libraries’ information literacy programs. The framework includes five generic dimensions, associates competences to each dimension, and translates these competencies into instructional topics to facilitate interpretation and direct implementation. The dimensions and respective competences are: (1) understanding data, i.e., general knowledge and awareness of data, how they are generated and what are the different types and sources of data; (2) finding and/or obtaining data, i.e., skills required to access/assess data sources; (3) reading, interpreting and evaluating data, i.e., competences relevant to presenting data and to critically evaluating them; (4) managing data, i.e., skills related to metadata data management repositories and data reuse; and (5) using data, i.e., skills and knowledge required to properly and ethically handle and synthesize data. Although the proposed scheme aspires to be universal, the key to its success lies in the depth to which it is developed, after adaptation to each library’s particular needs.

Finally, Marsh (2012) recognized the need to proactively foster the use of data to guide educational decision-making and practice and outlined an EDL competence model of five components of the data use process. The author conducted a review of related research, aiming to facilitate the understanding of what research tells us about interventions designed to support the process of educational data use and to identify where there are possible gaps. The framework targets at defining the dimensions or core features of these interventions, the types of data and data users that were targeted, the implementation of interventions, as well as their outcomes. The dimensions include: (1) accessing or collecting data, (2) filtering, organizing, or analyzing data into information, (3) combining information with expertise and understanding to build knowledge, (4) knowing how to respond and taking action or adjusting one's practice, and (5) assessing the effectiveness of these actions or outcomes that result.

Based on these EDL CFs, Table 1 demonstrates a synopsis and synthesis of EDL dimensions. According to these frameworks, the decisions that educators need to use educational data to inform are multiple and diverse, and educational data literacy is tailored to the specific use (context-aware) (Mandinach & Gummer, 2016).

**Table 1:** Identified EDL dimensions according to the EDL competence frameworks

	Identified EDL dimension	EDL CF^a^				
		1	2	3	4	5
Generic	Professionalism			X		
	Content-curriculum			X		
	Pedagogy			X		
	Question posing/identify problems	X		X		
Data-related	Data location/access/collection	X	X		X	X
	Data comprehension	X				X
	Data interpretation/transform to information	X		X	X	X
	Data use/application/act on	X	X	X	X	X
	Data analysis				X	
	Data evaluation		X	X	X	X
	Data management		X		X	X

^a^1. Means et al. (2011); 2. Ridsdale et al. (2015); 3. Mandinach and Gummer (2016); 4. Marsh (2012); 5.Prado and Marzal (2013)

Regarding the EDL-related courses, seven (7) of them were offered in higher education programs and the remaining five (5) were offered for supporting professional development. The courses were viewed and analysed with respect to their learning objectives, having in mind how they could launch EDL skills. Table 2 summarizes the objectives of the identified courses.

**Table 2:** Objectives of the identified professional development and higher education courses

Learning objectives	Professional development and higher education courses^a^											
	1	2	3	4	5	6	7	8	9	10	11	12
Understand and use data effectively					×				X			
Efficiently collect educational data and metadata from a wide range of data sources for future analyses										X	X	X
Manipulate the educational datasets that capture the learning experience										X		X
Conduct (basic) data wrangling and analyses					X	X	X	X		X		X
Employ teaching analytics to analyse the lesson plans				X								
Inform teaching and learning decisions				X					X		X	
Deploy personalized support actions for the students												X
Use data-driven methods to answer practical educational questions				X		X	X	X				
Address evaluation issues, key diagnostic metrics and their uses, and validity issues							X					
Reflect on the teaching practice by combining insights from both teaching and learning analytics				X								
Raise ethics and privacy considerations	X				X							
Other learning objectives (non data-related)	3	4	6	1	2	1	3	2	0	5	1	0

^a^1. Digital Competence (https://www.ntnu.edu/studies/courses/EDU3084#tab=omEmnet); 2. Digital Literacy—Smart Learning (https://www.ntnu.edu/studies/courses/SOS2020/2017/1#tab=omEmnet); 3. Data Literacy 01 (http://artofeducating.com/online-courses/); 4. Analytics for the Classroom Teacher (https://www.edx.org/course/analytics-for-the-classroom-teacher); 5. Learning Analytics Fundamentals (https://www.edx.org/course/learning-analytics-fundamentals-utarlingtonx-link-la-fundx); 6. Big Data and Education (https://www.edx.org/course/big-data-education-pennx-bde1x-0); 7. Data, Analytics and Learning (https://www.edx.org/course/data-analytics-learning-utarlingtonx-link5-10x); 8. Practical Learning Analytics (https://www.edx.org/course/practical-learning-analyticsmichiganx-plax); 9. Data Literacy for School Teachers—EDPZ6012 (https://sydney.edu.au/courses/units-of-study/2018/edpz/edpz6012.html); 10. Advancing computational and data literacy skills schools for life scientists (http://www.nhm.ac.uk/our-science/courses-and-students/advancing-computational-and-data-literacy-for-life-scientists.html); 11. Introduction to Data Wise: A Collaborative Process to Improve Learning & Teaching (https://www.edx.org/course/introduction-to-data-wise-a-collaborative-process?); 12. Using Data to Provide Personalized Student Support (https://www.edx.org/course/using-data-provide-personalized-student-utarlingtonx-link-la-ssax)

As seen from Table 2, the core objectives of these courses are: (a) to understand and use data effectively; (b) to efficiently collect educational data and metadata from a wide range of data sources for future analyses and to manipulate educational data-sets that capture the learning experience; (c) to conduct (basic) data wrangling and analyses; (d) to employ teaching analytics to analyse the lesson plans; (e) to inform teaching and learning decisions and to deploy personalized support actions for the students; (f) to use data-driven methods to answer practical educational questions; (g) to address evaluation issues, key diagnostic metrics and their uses, and validity issues, as well as to reflect on the teaching practice by combining insights from both teaching and learning analytics; (h) to raise ethics and privacy considerations. It should be noted that none of the identified courses was directly linked to any of the existing EDC CFs.

Furthermore, an important aspect in this process, highlighted in literature, is the ethical considerations that should be consistent throughout all phases of data manipulations (Data Quality Campaign, 2014; Wolff et al., 2016). These considerations typically include security, confidentiality, privacy, informed consent, and anonymity of peoples’ data. Although some authors put emphasis on data ethics, the majority fail to make note of it (Ifenthaler & Tracey, 2016). A recent review of the ethical issues in learning analytics (Tzimas & Demetriadis, 2021) demonstrate the shortage of empirical evidence-based guidelines on educational data analytics ethics and highlight the need to establish codes of practices to monitor and evaluate the respective ethics policies, so that trusted partners may use educational data analytics responsibly to improve teaching and learning. In order for IDs and eTUTs to understand and critically think about the larger issues regarding EDL, they must have an understanding and awareness of the ethics surrounding data (Ifenthaler & Tracey, 2016).

Since none of the existing EDL CFs provides explicit competence statements regarding the data-ethics dimension, we searched for more generic data-ethics frameworks that contain relevant statements. The search yielded six (6) frameworks (Clark et al., 2019; Clarke, 2018; Drew, 2018; HLEG AI, 2019; Tene & Polonetsky, 2016; Zook et al., 2017). All of them highlight the need to make use of informed consent when it has to do with collecting data from the subjects, and most of them focus on the protection of individuals’ privacy, confidentiality, integrity, security, authorship, and ownership. The issues of data governance, re-negotiation and data-sharing are also met on most frameworks.

### Dimensions of EDL and synthesis of the EDL-CF for instructional designers and e-tutors of online and blended courses

When it comes to determining EDL for IDs and eTUTs of online and blended courses in particular, the previous frameworks need to be further refined and adjusted accordingly. We argue that EDL for IDs and eTUTs should cover much more than the technical skills. As such, here we define Educational Data Literacy for IDs/eTUTs as *the ability to collect, manage, analyse, comprehend, interpret, and apply educational data in an ethical, meaningful, and critical manner*. This definition originates from the combined findings of the analysis of existing frameworks, enhanced with the competences deriving from the specific job roles, and focuses on informing the competence set which is required for digitally supported education professionals to give meaning to and act upon educational data from different sources, with the aim to continuously improve the teaching, learning, and assessment process, in an ethical way.

**Fig. 1:**
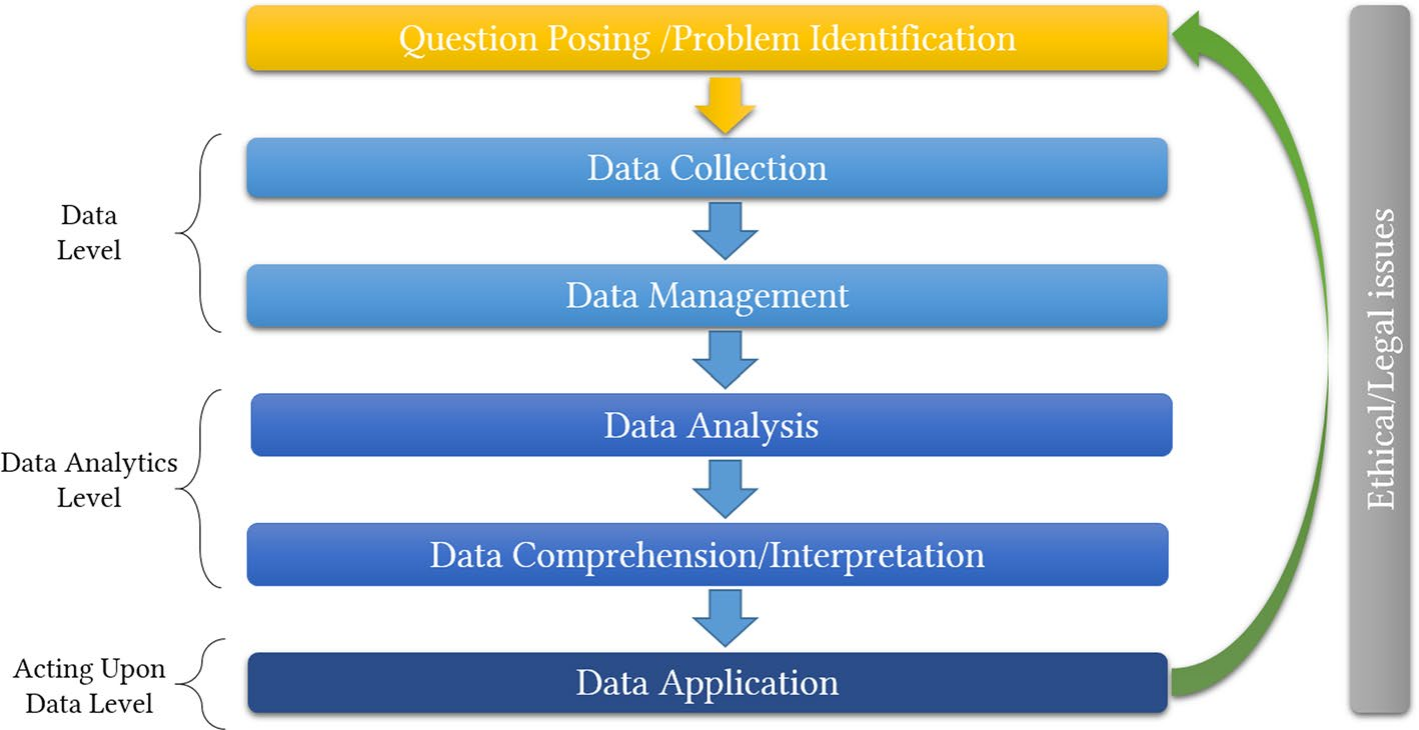
The EDL CF dimensions for the roles of ID/eTut

As a context-aware process, EDL is an inquiry cycle that involves: (a) data collection (e.g., location, discovery and access) and management (e.g., cleaning, organization and preservation) (*data level*), (b) data analysis (e.g., coding, analytics extraction, reporting on them), comprehension and interpretation (e.g., transforming the information from analytics into usable knowledge) (*data analytics level*), and (c) data application (e.g., deciding adaptations, providing feedback) (*acting upon data level*) to be used to inform instructional design of online/blended courses (ID), and to inform students’ guidance-support during online/blended courses (eTUT). In the beginning of the process, identifying the objectives/ problems and setting a purpose is required. In the broader definition of EDL and throughout the inquiry cycle, ethical and legal aspects catalyse the context-aware process cross-phase. This process is synopsized in Fig. 1.

Based on the above analyses of the dimensions and competence statements of the identified EDL frameworks and EDL-related courses, the consolidated EDL-CF for ID/eTUT consists of 1 generic and 5 data-related dimensions and 21 competence statements, synopsized in Table 3.

**Table 3:** The EDL-CF for ID and eTUT: dimensions and statements

EDL competence dimension	EDL competence statements
Data collection	Know where to find the right data/data sources
	Know how to obtain/access data
	Understand data quality and limitations (e.g., accuracy, completeness)
Data management	Identify the technologies to preserve data
	Know and apply data manipulation methods
	Know and apply data curation and data re-use methods
	Understand Data Description (Metadata)
Data analysis	Know and apply the basic data analysis methods
	Understand and apply the basic data analysis process steps
	Understand and apply the basic data presentation methods
Data comprehension & interpretation	Understand data (e.g., measurement error, discrepancies within data, key take-away points)
	Understand statistics
	Know how to interpret data (e.g., explanations of patterns, identification of hypotheses, connection of multiple observations)
	Generate potential connections to instruction
	Make decisions based on data
Data application	Use data to inform instruction
	Know how to share and cite data
	Evaluate the data-driven intervention
Data ethics	Explain the use of informed consent
	Know how to protect individuals' data privacy, confidentiality, integrity and security
	Understand authorship, ownership, data access (governance), re-negotiation and data-sharing

## Methodology

To validate the proposed EDL CP framework and identify areas of possible improvement, a study was conducted with *N* = 210 professionals with experience in digitally supported education from higher education institutes and elearning industry enterprises. An online survey instrument was designed to measure their perceptions regarding the current EDL readiness of IDs/eTUTs and the appropriateness of the proposed framework to map their profiles.

### Sampling

The selection of the appropriate participants was essential for the quality of our study. For that reason, the criteria used for their selection were their expertise on the field, their impact/ experience on the field, and diversity, i.e., selecting participants from different professional roles, geographic regions, and institution types (Iqbal & Pipon-Young, 2009). It was decided that the most appropriate participants for this study are professionals that are engaged in technology supported education and training, online and/or blended courses, or educational data literacy, as well as academics from the field of data science. The participants should be involved either in higher/secondary education or professional development, in various roles, namely, professional, academic, research, support, manager, leader, teacher. The targeted number of participants was 210, ideally covering the full range of professional roles in approximately equal numbers. In the recruitment process, around 150 professionals (i.e., university professors, secondary education teachers with a recognized interest in online/blended learning, designers of educational content) were initially contacted by the research group, directly through invitations over official channels and emails. The exact number of contacted professionals cannot be accurately estimated, since we also posted relevant invitations on professional group pages on social media (e.g., Facebook, LinkedIn). Finally, 210 professionals enrolled in the study. Descriptive statistics about the demographics of the participants (i.e., gender, age, geographical distribution, expertise in EDL, professional roles) are presented in “Descriptive statistics: analysis of participants profiles” section.

### Instruments

A questionnaire was developed in a web form (Google forms) to collect participants’ responses. The questionnaire consisted of 66 items in total and required approx. 60 min to be completed. After filling in the requested demographics (i.e., gender, age, country or region, definition of professional role, years involved in the role, years involved in the field of digital teaching and learning), the proposed EDL definition (see pages 13–14) and the framework in Table 3, consisting of the respective six (6) EDL dimensions and twenty-one (21) competence statements were presented and provided. Next, given the above definitions, the participants had to respond to 3 items regarding their perceptions on the EDL readiness level of ID/eTUT. Then, for each statement of each EDL dimension, a set of 3 (i.e., in total 63) was utilized for measuring (a) how well the statement addresses the specific dimension; (b) how important is the statement for IDs/eTUTs; and (c) how well written is the statement. All 66 items were answered in a 5-point Likert scale, where 1 stands for “strongly disagree”, and 5 stands for “strongly agree”. Specifically, the questionnaire consisted of the following items:

- ***Basic items (3) on Educational Data Literacy readiness of ID/eTUT***
  - I am familiar with the term Educational Data Literacy.
  - I believe that Instructional Designers and e-Tutors of Online and/or Blended Courses already possess Educational Data Literacy competences to a large extend.
  - I believe that Instructional Designers and e-Tutors of Online and/or Blended Courses need to possess Educational Data Literacy competences.
- ***For each statement (S***_***j***_***) of a given dimension (D***_***i***_***), (total 63 items type D***_***i***_***-S***_***j***_***)***
  - I believe that the EDL competence statement S_j_ addresses well the EDL competence dimension D_i_.
  - I believe that the EDL competence statement S_j_ is important for an Instructional Designer and an e-Tutor of Online and/or Blended Courses.
  - I believe that the EDL competence statement S_j_ is well written.

In addition, an invitation letter was sent to the professionals that agreed to participate in the study, along with guidelines for completing the survey in the right manner, as well as informing them about privacy and ethical issues. A consent form with all the information needed (purpose and procedure, potential benefits, potential risk or discomforts, storage of data, anonymity and confidentiality, right to withdraw, conflict of interest, compensation, participant concerns, and reporting) for the participants to consent or not in the survey, was also provided. The consent form follows the guidelines of the General Data Protection Regulation (EU) 679/2016 (‘GDPR’). The survey was conducted between 1st September to 15th October 2018.

### Data analysis

To gain a holistic view of the appropriateness of the sample across all anticipated demographic elements, descriptive statistics were employed. For exploring the participants’ familiarity with the concept of EDL, their responses were analysed using cross-tabulation, whilst their opinions about the EDL-readiness of and usefulness for ID/eTUT were analysed using frequencies.

Furthermore, for the validation of the appropriateness of the proposed framework—measured with the three items used for assessing the 21 competence statements of the 6 dimensions—validity analysis (i.e., Content Validity Index—CVI) and criterion validity (i.e., Spearman’s Correlation coefficient) were employed. In addition, factor analysis was also performed. The Keiser-Meyer-Olkin index was employed to evaluate the sufficiency of the sample (i.e., should be higher than 0.5). All analyses were conducted using SPSS v. 25 for Windows.

## Results

### Descriptive statistics: analysis of participants profiles

Descriptive statistics were used to confirm the distribution of participants across all anticipated demographic elements. Specifically, in relation to:

- the **gender**, the distribution of the participants is relatively balanced with 32.86% female and 66.19% male participants;
- the **age**, the majority of participants (36.2%) are between 40 and 50, with 86.68% being between 30 and 60 years old (*M* = 43.65 years, *SD* = 10.05 years);
- the **geographical distribution**, the participants were from 31 different countries with 17 of them being EU members. Most of participants were from Europe (75.24%), with a fair representation of other continents (America [12.86%], Asia–Pacific [11.9%]);
- the **level of professional experience**, most participants can be considered experienced in their professional roles (73.80% more than 6 years) and in the field of digital teaching and learning (80.5% more than 6 years). Most participants in both categories reported 11–20 years of experience (i.e., in their professional roles: *M* = 1.65, *SD* = 7.76, *N* = 69; in the field of digital teaching and learning: *M* = 13.27, *SD* = 7.77, *N* = 84);
- **expertise in EDL** according to their professional role, 88.10% has a reasonable level of understanding of EDL and 40.50% have a high-level of expertise in EDL.

This is a strong evidence that the level of professional experience and the level of EDL expertise of the participants demonstrate the reliability of the sample.

### EDL competence usefulness for and readiness of ID/eTUT

Next, we analysed the participants’ responses to the three corresponding items about EDL-readiness of ID/eTUT.

First, the *level of familiarity* with the concept of EDL was examined. Most of the participants (69.52%) identified themselves as being familiar with this concept (i.e., scored either 4 or 5). Then, we cross examined these responses with respect to participants’ self-reported expertise (professional role). Most of the participants identified themselves as “Experts”, i.e., self-reported as “Experts with Experience in EDL” in the demographics question, (91.20%) claiming that they are familiar with the term Educational Data Literacy (scores 5, strongly agree and 4, agree), as expected. Three participants identified themselves with low familiarity (score 2) with the term EDL and thus can be considered as outliers. Lastly, a substantial percentage (65.34%) of “Non-Experts”, i.e., self-reported as something else except from “Experts with Experience in EDL” in the demographics question, were familiar with the term EDL, giving a score of 4 (32.38%) or 5 (32.95%).

Next, we examined the participants’ opinion about the *EDL competence readiness* of ID/eTUT. Most of the participants (75.24%) scored 3 or less, indicating that they consider the EDL competence readiness of ID/eTUT as *not adequate*, although a considerable percentage (24.76%) declared otherwise. We further analysed the same issue for the two expertise-based subgroups of the participants (i.e., “Experts” and “Non-Experts”, as previously). As expected, most of the “Experts” (91.18%) scored 3 or less, indicating that they consider ID/eTUT not to be EDL-competence ready. Quite similarly, most of “Non-Experts” (72.16%) also believe that ID/eTUT need to gain EDL competence. This finding indicates that all participants demonstrate an overwhelming confidence that ID/eTUT are missing or have limited EDL competence.

We also examined *how useful* the participants perceive that EDL competences are for ID/eTUT. The analysis was conducted for the sample as a whole and for the two sub-groups of “Experts” and “Non-Experts”, as previously. Most of the total sample (89.53%) demonstrated an overwhelming confidence that EDL competences are useful for ID/eTUT (scored 4 or 5), highlighting the importance of those competences for the digital teaching and learning professionals. Specifically, 91.18% from the “Experts” sub-group consider essential the EDL competence for ID/eTUT (scored 4 or 5), whilst slightly less (89.20%) are the “Non-Experts” who agree with that opinion. The above results are synopsized in Table 4.

**Table 4:** Opinions about EDL readiness of and usefulness for ID/eTUT

	Total sample (score > 3) (%)	EDL experts (score > 3) (%)	EDL non-experts (score > 3) (%)
Level of familiarity with EDL	69.52	91.20	65.34
EDL competences readiness	24.76	8.82	27.84
EDL competences usefulness	89.53	91.18	89.20

### Content validity

Content validity is the degree to which an instrument has an appropriate sample of items for the construct being measured, i.e., it refers to the extent to which a measure represents all facets of a given construct and is an important procedure in scale development. Content Validity Index (CVI) is the most widely used index in quantitative evaluation. There are two kinds of CVI: I-CVI and S-CVI/Ave. The first type involves the content validity of individual items and the second involves the content validity of the overall scale (the average).

Content validity of individual items, I-CVI index, refers to the proportion of content experts giving item (Q_i_, i = 1, 2, 3) a relevance rating of 4 or 5. Values of I-CVI range from 0 to 1, with I-CVI > 0.76, the item being relevant, between 0.70 and 0.76, the item needs revisions, and if the value is below 0.70, the item should be eliminated. Researchers recommend that a scale with excellent content validity should be composed of I-CVIs of 0.76 or higher and S-CVI/Ave of 0.78 or higher, respectively. For establishing Content Validity, the I-CVI index was calculated by dividing the number of the responders that graded with 4 or 5 (thus dichotomizing the ordinal scale into “agree”, “strongly agree” and “disagree”, “strongly disagree”) by the total number of the responders. Additionally, S-CVI/Ave averages the proportion of items rated 4 or 5 across the responders. Tables 5 and 6 synopsize the results for I-CVI and S-CVI/Ave respectively, for the 210 participants for every item in all statements and all dimensions.

**Table 5:** I-CVI for every item in all statements in all dimensions

	D_1_	D_2_	D_3_	D_4_	D_5_	D_6_
S_1_						
Q_1_	0.87	0.80	0.87	0.84	0.89	0.91
Q_2_	0.87	**0.70**	0.83	0.83	0.92	0.87
Q_3_	0.79	**0.70**	0.76	**0.71**	0.78	0.80
S_2_						
Q_1_	0.84	0.83	0.81	0.76	0.86	0.93
Q_2_	0.87	**0.74**	0.78	0.79	0.79	0.94
Q_3_	0.81	**0.75**	**0.67**	**0.62**	0.81	0.88
S_3_						
Q_1_	0.86	0.80	0.87	0.89	0.84	0.92
Q_2_	0.91	**0.73**	0.87	0.88	0.82	0.90
Q_3_	0.80	**0.75**	0.77	0.82	0.76	0.84
S_4_						
Q_1_		0.83		**0.74**		
Q_2_		0.80		0.81		
Q_3_		0.76		**0.68**		
S_5_						
Q_1_				0.87		
Q_2_				0.91		
Q_3_				0.80

**Table 6:** S-CVI/Ave for all statements in all dimensions

	D_1_	D_2_	D_3_	D_4_	D_5_	D_6_
S_1_	0.84	**0.74**	0.82	0.80	0.86	0.86
S_2_	0.84	0.78	**0.75**	**0.72**	0.82	0.92
S_3_	0.86	**0.76**	0.84	0.87	0.80	0.89
S_4_		0.80		**0.74**		
S_5_				0.86		

Specifically, as seen from Tables 5 and 6, for dimensions D_1_, D_5_, D_6_, all values for the I-CVI index are over 0.76, and all values of S-CVI/Ave are over 0.78, providing evidence for the validity of each statement in these dimensions.

However, for dimension D_2_, the I-CVI index appears to be on the boundary of the accept level (0.7) for both items Q_2_ and Q_3_ for statement S_1_, resulting in the lowest value of S-CVI/Ave on this dimension. In addition, for both items Q_2_ and Q_3_ for statements S_2_ and S_3_, I-CVI is lower than 0.76, which implies further exploration is also needed.

For dimension D_3_, I-CVI score is high in every Q_i_ except for S_2_Q_3_ which is less than 0.7. This is an indication that the statement may be needing more exploration. We see that S-CVI/Ave is affected by this phenomenon resulting is low score in the statement S_3_.

Moving to dimension D_4_, for statements S_2_ and S_4_, the I-CVI was below the acceptable value (i.e., 0.7) for item Q_3_ and S-CVI/Ave was below 0.76 for both statements. Another noticeable result is the scores of S_1_Q_3_, S_4_Q_1_ which appear to be near the lower acceptance level. The rest of the items Q_1_, Q_2_, Q_3_ in every statement produce a high score of I-CVI index. The low values of the I-CVI index of item Q_3_ for statements S_2_ and S_4_ influence the S-CVI/Ave strongly producing the score of 0.72 for S_2_ and 0.74 for S_4_.

### Criterion validity

We also examined the criterion validity of the questionnaire using Spearman’s rho Correlation coefficient, i.e., by correlating each questionnaire item’s scores with the total score. Items’ scores that are significantly correlated with the total score indicate that the items are valid. For all dimensions except D_4_, the correlation coefficient of each item was very high, in accordance to the total score in each statement as well as with the rest of the items. Furthermore the sig. value (2-tailed) was of 0.001 < 0.05, and therefore we can conclude that the respective items are valid. Regarding the correlation coefficient in dimension D_4_, there is a lower value (0.665) in comparison with the total score. Furthermore, the correlation coefficient of D_4_S_2_Q_2_–D_4_S_2_Q_3_ is low. Overall because the Spearman’s rho in relation with the total score is higher than 0.3, the items in D_4_ are also valid. The full table of correlations can be found in “Appendix” (Table 8).

Furthermore, internal consistency was also evaluated by examining the correlations among each of the items using Pearson’s r correlations coefficient, which quantifies the strength of the relationship between the variables. Pearson’s test suggests all the items are relatively strong related. In particular, the inter-items correlation coefficients varied from 0.33 (D_4_S_4_ and D_3_S_2_) to 0.78 (D_6_S_3_ and D_6_S_2_), can be seen in detail in Table 9 in the “Appendix”.

### Factor analysis

Factor analysis and especially exploratory factor analysis allows to test the hypothesis that a relationship between the observed variables and their underlying latent construct exists. Based on the results from content validity (Table 5), we excluded item Q_3_ from statements D3S2, D_4_S_2_ and D_4_S_4_ because it mostly threatened the validity of our instrument. The remaining items were considered as variables on which we applied Principal Component Analysis (PCA).

We applied PCA using the traditional cut-off of an eigenvalue of 1.0. The screeplot indicated eight factors. Since rotations minimize the complexity of the factor loadings to make the structure simpler to interpret, and due to the fact that factor loading matrices are not unique, i.e., for any solution involving two or more factors there are an infinite number of orientations of the factors that explain the original data equally well, we performed Promax rotation because the individual factors were correlated (Yong & Pearce, 2013). Each rotated loading matrix was examined for items that did not load (i.e., their loading value was lower than |0.3|) or cross-loaded (i.e., having two or more values that loaded above |0.30| in multiple factors (Tabachnick, & Fidell, 2007)). After cross-loading items were identified, they were removed from the factor analysis and the factor analysis was performed again. This process (examining for non-loading and cross-loading questions) was completed until a solution was found that was free of non-loading and cross-loading items. Through this process, six factors were retained from items loading onto a single factor with a value greater than |0.30| and no cross-loading items. The solution is illustrated in Table 7. Furthermore, the Keiser–Meyer–Olkin index that evaluates the sufficiency of the sample (greater than 0.5), and Barlett’s Test of Sphericity that evaluates if the correlations of our items allow to apply factor analysis (sig. value < 0.05) were satisfied.

**Table 7:** Factor analysis

Pattern matrix^a^						
	Component					
	1	2	3	4	5	6
D_1_S_1_Q_2_	0.031	− 0.084	**0.858**	− 0.019	0.063	0.054
D_1_S_2_Q_2_	0.004	− 0.013	**0.868**	− 0.060	0.161	− 0.071
D_1_S_3_Q_2_	0.023	0.109	**0.777**	0.031	− 0.070	− 0.013
D_2_S_1_Q_2_	− 0.073	− 0.057	0.054	0.041	**0.678**	0.219
D_2_S_2_Q_2_	− 0.052	0.115	0.052	0.180	**0.750**	− 0.092
D_2_S_3_Q_2_	0.059	0.149	0.109	− 0.142	**0.695**	0.125
D_2_S_4_Q_1_	**0.555**	0.098	0.114	0.261	− 0.083	− 0.056
D_3_S_1_Q_2_	− 0.058	**0.804**	0.060	0.047	0.108	− 0.117
D_3_S_2_Q_2_	− 0.036	**0.725**	0.008	0.053	0.264	− 0.143
D_3_S_3_Q_2_	0.120	**0.906**	− 0.070	− 0.203	− 0.049	0.132
D_4_S_1_Q_2_	− 0.172	0.220	0.056	**0.463**	− 0.242	0.286
D_4_S_2_Q_1_	**0.891**	− 0.092	− 0.049	− 0.055	0.031	− 0.042
D_4_S_3_Q_1_	**0.624**	0.069	− 0.008	0.248	− 0.127	0.021
D_4_S_4_Q_2_	− 0.052	− 0.091	0.042	**0.924**	− 0.043	− 0.009
D_4_S_5_Q_1_	**0.760**	− 0.015	0.108	0.066	− 0.110	0.034
D_5_S_1_Q_2_	0.146	− 0.048	0.036	**0.650**	0.152	− 0.054
D_5_S_2_Q_1_	**0.800**	0.075	− 0.031	− 0.190	0.120	0.100
D_5_S_3_Q_2_	0.110	− 0.026	− 0.270	**0.630**	0.297	− 0.023
D_6_S_1_Q_2_	− 0.053	− 0.229	0.121	0.269	0.130	**0.615**
D_6_S_2_Q_2_	0.125	− 0.032	− 0.048	− 0.180	0.068	**0.905**
D_6_S_3_Q_2_	− 0.055	0.149	− 0.052	0.071	0.054	**0.765**

Extraction method: Principal Component Analysis

Rotation method: Promax with Kaiser Normalization

^a^Rotation converged in 7 iterations

In this table, the values in bold show which items (rows) loads on the respecive factor (columns)

Again, from Table 7, it becomes apparent that the previously identified as problematic items, indeed do not load on the factor that corresponds to the respective EDL dimension, making it explicitly clear, that those items require revision. Overall, this analysis suggests that the structure of the proposed EDL framework is stable for the sample of *N* = 210 professionals.

## Discussion

The present work demonstrated the design, development, and validation processes of an EDL CP framework for IDs/eTUTs. The framework aims at addressing the challenges raising from educational data with a goal to support teaching and learning professionals in data-informed decision making by enhancing their educational data literacy. The protocol for developing a sound framework had as initial task the environmental scan for relevant frameworks and professional development courses. The literature scan yielded 5 relevant frameworks and 12 courses that were further analyzed. No previous framework for the roles of IDs/eTUTs was identified. Furthermore, as seen in Table 1, none of the existing frameworks covers all data-relevant issues, with most of the approaches consisting of four to six dimensions and raising data-oriented issues from specific perspectives. Similarly, as illustrated in Table 2, the data-related objectives the courses attempt to address vary between 1 and 4, with most of the courses to include other learning objectives, as well, beyond the data-oriented ones.

Furthermore, in some cases, the identified dimensions either merged data-related processes that, from a data-expert perspective, are distinct (e.g., ‘*data evaluation, i.e., skills related to data analysis, presentation, interpretation and to making instructional decisions from data*’ (Ridsdale et al., 2015)), or introduced dimensions that can be considered as very generic (e.g., ‘*use data*’ (Mandinach & Gummer, 2016; Prado & Marzal, 2013)), or were lacking essential data-related competences (e.g., data analysis is missing (Means et al., 2011)), or did not provide explicit competences but were focusing only on the dimensions themselves (Marsh, 2012). In previous work, it has been emphasized that most approaches to educators’ data literacy tend to cover fragmented sets of abilities, and address technical skills, with less emphasis on critical, ethical and personal approaches to datafication in education (Raffaghelli & Stewart, 2020). More importantly, specialized training of teachers, that will help them to attain an adequate level of data literacy has been acknowledges as essential towards facing the challenges of the post-pandemic era (Sánchez-Cruzado et al., 2021).

Based on the yielded literature search results, and by combining the above findings, we identified 5 dimensions of EDL and 18 statements to consider in the suggested framework. In addition, and given the increased attention on ethical aspects of data usage, 6 generic data-ethics frameworks that contain relevant statements were analyzed, resulting in one more dimension with three associated statements about the ethical aspects of data.

Next, the proposal was empirically evaluated both from the aspect of EDL-competence readiness and usefulness, and from the appropriateness of the dimensions and statements considered, with the participation of *N* = 210 professionals in digitally supported education. The profiles of the participants were evaluated, and the analysis confirmed the distribution of professionals across all anticipated demographic elements.

Based on the analysis of the responses, most of the participants consider the EDL competence readiness of ID/eTUT as not adequate, with a high percentage (91.18%) of the ‘EDL-experts’, i.e., participants with expertise in EDL, denoting that ID/eTUT are not yet EDL-competent ready. In accordance with this finding, the majority of the sample (89.53%) demonstrated an overwhelming confidence that EDL competences are useful for ID/eTUT, highlighting the importance of those competences for the digital teaching and learning professionals, and acknowledging the urgency for their development.

Furthermore, in relation to the validation of the proposed EDL CP framework, our findings indicate that the participants agree with most of the considered competence statements in terms of how well they address the corresponding EDL dimensions and how important they are to the dimensions they belong to, suggesting however, that slight improvements are necessary in some of them, on the way the statements are written. Specifically, content validity (i.e., the extent to which a measure represents all facets of a given construct) was confirmed for all items of dimensions 1, 5 and 6 (I-CVI higher than 0.76 and S-CVI/Ave higher than 0.78). However, this analysis shown that statements S_1_ ‘*Identify the technologies to preserve data*’ and S_3_ ‘*Know and apply data curation and data re-use methods*’ in dimension D_2_, as well as statement S_2_ ‘*Understand and apply the basic data analysis process steps*’ in dimension D_3_ and statements S_2_ ‘*Understand statistics*’ and S_4_ ‘*Generate potential connections to instruction*’ in dimensions D_4_ require attention and the items need revisions. Spearman’s rho correlation coefficient also shown that statement S_2_ of dimension D_4_ is problematic (*ρ* = 0.252).

Next, to test the hypothesis that a relationship between the observed variables and their underlying latent construct exists, we performed Exploratory Factor Analysis. Specifically, after iteratively removing the non-loading and the cross-loading items, we repeatedly performed Principal Component Analysis with Promax rotation, and concluded to a six factors model. As seen from Table 7, while some items loaded onto different factors than in the initially proposed framework, in general, the items were similarly grouped, and in line with the EDL dimensions. One can notice that the items that were ‘misclassified’ were the ones that from the previous analyses were identified as potentially problematic and require revisions.

### Limitations

Despite the promising findings concerning the evaluation of the proposed EDL competence profiles framework, the work presented in this paper has also some limitations. Firstly, since the topic of EDL has emerged recently, we employed scoping review as the methodological approach to scan related literature. This method does not appraise the quality of evidence in the primary research reports in any formal sense. The framework presented here is a first robust attempt to define the dimensions and statements of the EDL profiles, yet, once more empirical evidence on the topic becomes available, the recommended framework needs to be revised accordingly. Secondly, the framework was designed and evaluated before the Covid-19 pandemic. However, the pandemic has revealed the real-world practical needs of IDs/eTUTs with respect to their educational data literacy. Developing an instrument for collecting the beliefs and expectations of digitally supported educational professionals will provide insights that can significantly improve the framework, mostly regarding the readiness of the professionals. Lastly, the performed analyses of the professionals’ responses show that the structure proposed to frame the development and acquisition of EDL competences is overall stable, yet some of the considered statements require further attention, exploration, and revision. This is within our current work plans.

## Conclusions

As informing and improving everyday decisions based on available data is rapidly becoming a commonplace (Mortier et al., 2014), and the need to acquire data literacy competences is gaining momentum (Shields, 2005), it is also a one-way path to develop sustainable frameworks and to invest on solid foundations for the development of these competences. Particularly for the professionals in the domain of digital teaching and learning, the rise of learning and teaching analytics has influenced the way instructional designers and e-trainers are making data-informed educational decisions (Ifenthaler et al., 2018), and has opened the discussions for reshaping the educational data literacy landscape (Wasson et al., 2016). Educational Data Literacy (EDL) is conceptualized as the ability to collect, manage, analyse, comprehend, interpret, and apply upon educational data in an ethical, meaningful, and critical manner.

In professional learning and teaching contexts, the design of professional development courses and the construction of instruments for competence assessment or course accreditation in professional learning and teaching can be facilitated through a set of skills, knowledge, and attitudes—in general competences—that are possessed or needed to be acquired by the professionals within the specific context (Sampson & Fytros, 2008). Several competence frameworks have been proposed and have succeeded to create a common language in terms of competences for instructional designers and e-trainers (e.g., CEN, 2016; Mishra & Koehler, 2006; UNESCO, 2011; Wakefield, Warren & Mills, 2012). However, these frameworks face a common significant shortcoming: they primarily aim to upskill professionals in terms of educational design concepts and processes, or generic ICT competences and digital literacy; they scarcely—if at all—accommodate emerging advancements in the field of digital learning related to the use of educational data analytics methods (Sergis & Sampson, 2017) and the need for Educational Data Literacy (EDL). Research on the topic of data literacy for educators has been characterized as sporadic and scarce; it is essential to support educators acquire data related competences (from setting a purpose to use data, to collecting, analyzing, and interpreting the data, and taking instructional action) so that they can prevent ill-informed decisions that can significantly affect students (Kippers et al., 2018; Ndukwe & Daniel, 2020). Furthermore, several frameworks have been proposed the past decade for addressing issues related to data literacy competences—some of those frameworks originate from areas that are close to the educational domain (e.g., library studies), while others focus on data literacy for teaching (e.g., Mandinach & Gummer, 2016; Prado & Marzal, 2013; Ridsdale et al., 2015). Despite the fact that these proposals aim to tackle the same/similar problem(s), the diversity in their approaches leads to heterogeneous competences descriptions that cannot be formally described and represented in a unified manner, and none of those frameworks focuses on the CPs of IDs/eTUTs of online and blended courses. It has been highlighted that less emphasis has been given on critical, ethical and personal approaches to datafication in education (Raffaghelli & Stewart, 2020), and that training educational professionals on acquiring data literacy competences is essential on the way to digitalized education (Henderson, & Corry, 2020; Sánchez-Cruzado et al., 2021).

This study proposed an EDL CP framework and evaluated its soundness by analysing the responses of *N* = 210 professionals with experience in digitally supported education from higher education institutes and elearning industry enterprises. The framework was designed following a scoping review methodology, that included both a review of literature and a scan of related courses. The ethical dimension of data has also been considered. From the evaluation of the framework, the participants agreed on the usefulness of EDL competences for ID/eTUT and believe that the digital learning and teaching professionals are not EDL-competent to date. This finding is in line with previous results (e.g., Kippers et al., 2018; Ndukwe & Daniel, 2020; Reeves & Chiang, 2019) and thus, this study contributes to literature with a framework that can be considered as a first robust approach to constitute the solid ground to guide the design of courses targeting at the development of IDs’/eTUTs’ data literacy.

## Data Availability

The datasets used and/or analysed during the current study are available from the corresponding author on reasonable request.

## Appendix

See Tables 8 and 9.

**Table 8:** Spearman’s rho

Spearman's rho	totalD1S1	Sig. (2-tailed)	D1S1Q1	D1S1Q2	D1S1Q3
D1S1Q1	.806**	.000	1.000	.526**	.588**
D1S1Q2	.807**	.000		1.000	.533**
D1S1Q3	.868**	.000			1.000
D1S2Q1	.872**	.000	1.000	.610**	.742**
D1S2Q2	.833**	.000		1.000	.604**
D1S2Q3	.900**	.000			1.000
D1S3Q1	.842**	.000	1.000	.530**	.705**
D1S3Q2	.768**	.000		1.000	.569**
D1S3Q3	.918**	.000			1.000
D2S1Q1	.853**	.000	1.000	.525**	.670**
D2S1Q2	.793**	.000		1.000	.447**
D2S1Q3	.845**	.000			1.000
D2S2Q1	.821**	.000	1.000	.458**	.583**
D2S2Q2	.773**	.000		1.000	.387**
D2S2Q3	.824**	.000			1.000
D2S3Q1	.855**	.000	1.000	.547**	.688**
D2S3Q2	.794**	.000		1.000	.452**
D2S3Q3	.854**	.000			1.000
D2S4Q1	.845**	.000	1.000	.561**	.676**
D2S4Q2	.820**	.000		1.000	.541**
D2S4Q3	.871**	.000			1.000
D3S1Q1	.841**	.000	1.000	.456**	.627**
D3S1Q2	.750**	.000		1.000	.404**
D3S1Q3	.851**	.000			1.000
D3S2Q1	.830**	.000	1.000	.521**	.680**
D3S2Q2	.762^**^	.000		1.000	.504**
D3S2Q3	.902**	.000			1.000
D3S3Q1	.827**	.000	1.000	.475**	.653**
D3S3Q2	.694**	.000		1.000	.357**
D3S3Q3	.874*	.000			1.000
D4S1Q1	.882**	.000	1.000	.512**	.709**
D4S1Q2	.751**	.000		1.000	.418**
D4S1Q3	.870**	.000			1.000
D4S2Q1	.835**	.000	1.000	.490**	.577**
D4S2Q2	.665**	.000		1.000	.252**
D4S2Q3	.829**	.000			1.000
D4S3Q1	.854**	.000	1.000	.541**	.630**
D4S3Q2	.790**	.000		1.000	.428**
D4S3Q3	.844**	.000			1.000
D4S4Q1	.912**	.000	1.000	.696**	.774**
D4S4Q2	.827**	.000		1.000	.629**
D4S4Q3	.919**	.000			1.000
D4S5Q1	.887**	.000	1.000	.449**	.801**
D4S5Q2	.744**	.000		1.000	.488**
D4S5Q3	.912**	.000			1.000

**Table 9:** Inter-item correlation coefficients

Inter-item correlation matrix																						
	M (SD)	D1S1	D1S2	D1S3	D2S1	D2S2	D2S3	D2S4	D3S1	D3S2	D3S3	D4S1	D4S2	D4S3	D4S4	D4S5	D5S1	D5S2	D5S3	D6S1	D6S2	D6S3
D1S1	4.24 (0.67)	1.00																				
D1S2	4.27 (0.69)	0.71	1.00																			
D1S3	4.32 (0.69)	0.64	0.60	1.00																		
D2S1	4.03 (0.77)	0.56	0.48	0.49	1.00																	
D2S2	4.13 (0.73)	0.56	0.54	0.55	0.65	1.00																
D2S3	4.09 (0.77)	0.58	0.56	0.62	0.62	0.73	1.00															
D2S4	4.23 (0.74)	0.55	0.52	0.55	0.49	0.56	0.57	1.00														
D3S1	4.28 (0.75)	0.51	0.51	0.60	0.40	0.49	0.52	0.56	1.00													
D3S2	4.11 (0.85)	0.41	0.50	0.49	0.39	0.50	0.53	0.56	0.70	1.00												
D3S3	4.32 (0.69)	0.48	0.52	0.58	0.43	0.50	0.54	0.62	0.76	0.68	1.00											
D4S1	4.21 (0.78)	0.49	0.43	0.56	0.46	0.47	0.47	0.52	0.48	0.51	0.52	1.00										
D4S2	4.04 (0.78)	0.48	0.49	0.50	0.45	0.53	0.53	0.53	0.52	0.50	0.52	0.55	1.00									
D4S3	4.40 (0.69)	0.55	0.49	0.59	0.49	0.53	0.59	0.59	0.56	0.55	0.57	0.66	0.62	1.00								
D4S4	4.13 (0.91)	0.51	0.43	0.45	0.51	0.44	0.45	0.57	0.37	0.33	0.40	0.47	0.42	0.54	1.00							
D4S5	4.41 (0.67)	0.61	0.60	0.55	0.48	0.49	0.45	0.54	0.55	0.48	0.55	0.49	0.58	0.60	0.54	1.00						
D5S1	4.36 (0.70)	0.56	0.48	0.53	0.60	0.62	0.50	0.53	0.43	0.42	0.49	0.45	0.51	0.55	0.55	0.57	1.00					
D5S2	4.31 (0.77)	0.49	0.52	0.53	0.48	0.52	0.59	0.54	0.45	0.46	0.58	0.48	0.56	0.59	0.36	0.52	0.53	1.00				
D5S3	4.24 (0.78)	0.49	0.52	0.50	0.51	0.55	0.58	0.56	0.49	0.48	0.46	0.50	0.49	0.64	0.54	0.60	0.53	0.54	1.00			
D6S1	4.41 (0.69)	0.52	0.51	0.52	0.47	0.50	0.52	0.51	0.46	0.46	0.44	0.50	0.46	0.56	0.48	0.56	0.52	0.46	0.51	1.00		
D6S2	4.57 (0.57)	0.58	0.48	0.62	0.49	0.51	0.55	0.53	0.47	0.46	0.50	0.57	0.51	0.65	0.42	0.53	0.50	0.60	0.45	0.60	1.00	
D6S3	4.48 (0.63)	0.51	0.48	0.52	0.47	0.51	0.55	0.61	0.43	0.45	0.49	0.60	0.46	0.63	0.40	0.44	0.44	0.61	0.48	0.59	0.78	1.00

## Abbreviations

CP: competence profile
CVI: content validity index
DL: data literacy
EDL: educational data literacy
EDL CF: educational data literacy competence framework
EDL CP: educational data literacy competence profiles
eTUT: e-tutor
ID: instructional designer
LMS: learning management system
PCA: principal component analysis
